# The impact of intellectual property demonstration policies on carbon emission efficiency

**DOI:** 10.1038/s41598-024-66372-8

**Published:** 2024-07-03

**Authors:** Lu Yao, Aoyu Li, Shuhua Wang

**Affiliations:** 1 School of Economics and Management, Taiyuan Normal University, Jinzhong, China; 2 School of Software, Taiyuan University of Technology, Taiyuan, China; 3 Institute of Shanxi Merchant Studies, Shanxi University of Finance and Economics, Taiyuan, China

**Keywords:** Intellectual property demonstration policy, Carbon emission efficiency, Green technological innovation, Industrial structure upgrading, Spatial spillover, Climate-change mitigation, Climate-change policy, Environmental economics

## Abstract

Confronted with the concurrent challenges of economic advancement and environmental management, this study explores whether implementing Intellectual Property Demonstration Policies (IPDP) can be a covert force in enhancing carbon emission efficiency. Utilizing panel data from 280 prefecture-level cities in China over the period 2007–2019, we employ a quasi-natural experimental design, incorporating multiple-period difference-in-differences models, mediation effect models, and spatial Durbin difference-in-differences models to assess the impacts of IPDP on carbon emission efficiency, its mechanisms of action, and its spatial spillover effects. The regression results of the multi-period difference-in-differences model reveal a statistically significant enhancement in carbon emission efficiency due to IPDP, with an impact coefficient of 0.044. Through heterogeneity tests, it is observed that the influence of IPDP on carbon emission efficiency varies based on regional characteristics, carbon emission levels, and the extent of marketization. The mediation effect model demonstrates that IPDP enhances carbon emission efficiency by fostering green technological innovation and facilitating the transformation of industrial structures. Furthermore, the spatial Durbin difference-in-differences model illustrates that IPDP positively influences the carbon emission efficiency of neighboring regions, indicating favorable spatial spillover effects. Notably, the indirect effect coefficients in the geographical distance matrix, economic distance matrix, and economic-geographical nested matrix are calculated as 0.673, 0.250, and 0.386, respectively. These findings offer compelling theoretical and empirical support for strengthening the intellectual property framework to optimize its environmental impact.

## Introduction

Accompanied by the rapid development of industrialization and urbanization, environmental quality has paid a tremendous price^1–3^. The substantial increase in global energy consumption has given rise to issues such as global warming and environmental deterioration, which have had severe repercussions on human health and societal well-being^4–7^. Nations worldwide have prioritized the reduction of carbon emissions as a fundamental measure to enhance environmental conditions^8–11^. As a responsible and dedicated developing country, China actively engages in global environmental management efforts. During the 75th United Nations General Assembly in 2020, China introduced the concept of dual carbon objectives, aiming to concurrently promote carbon reduction, pollution control, expansion of green initiatives, and economic growth. By 2022, China's carbon dioxide emissions had reached 11.48 billion tons, constituting nearly one-third of the global total, positioning China as a crucial arena for advancing carbon efficiency and reduction efforts.

Under the increasingly severe climate crisis, China is tasked with achieving peak carbon emissions by 2030 and carbon neutrality by 2060. To reach these goals, exploring new pathways to enhance carbon emission efficiency has become a crucial foundation for promoting high-quality economic development. Compared to total carbon emissions, carbon emission efficiency, viewed from an input–output perspective, can comprehensively reflect the costs of carbon emissions and the coupling relationship between resource-environment and socio-economic factors. Previous studies have investigated the determinants of carbon emission efficiency using conventional approaches, such as financial development^12,13^, technological innovation^14,15^, digital economy^16,17^, and industrial structure^18,19^. However, within the new development framework and the introduction of the "30·60 dual carbon" goals, the transition to a green and low-carbon economy imposes heightened demands on carbon reduction strategies, emphasizing institutional innovation as a critical and pressing task in the current establishment of an ecological civilization. Current research on reducing carbon emissions through institutional innovation tools primarily focuses on the influence of environmental policies on carbon emissions, such as low-carbon city pilots^20,21^, carbon emission trading rights^22,23^, and energy use rights trading policies^24^. Nevertheless, studies are scarce on the effective carbon reduction potential of intellectual property demonstration policies. Therefore, a systematic assessment of the impact of intellectual property demonstration policies on carbon efficiency serves as the fundamental starting point and focal point of this research.

The Intellectual Property Demonstration Policies (IPDP) represent a notable instance of institutional innovation designed to advance the holistic development of demonstration cities within a multidimensional framework that encompasses intellectual property generation (to foster innovation), utilization (to facilitate technology transfer), protection (to ensure property rights), management (to offer patent analysis), and services (to improve service quality). Since the inception of China's intellectual property (IP) strategy in 2007, the publication of the “National Intellectual Property Strategy Outline” in 2008, and the establishment of evaluation methodologies in 2011, 77 cities had been designated as National Intellectual Property Demonstration Cities by 2019. The principal objective of this policy is to reinforce IP protection, thereby mitigating the risks associated with the replication of new technologies and safeguarding corporate interests, ultimately facilitating the efficient allocation of production resources and stimulating corporate innovation. Notably, the IPDP has also played a pivotal role in enhancing carbon emission efficiency, creating new avenues for innovation in this sector. Consequently, investigating how this policy can effectively improve carbon emission efficiency emerges as a critical research priority amid the ongoing transformations in the realm of intellectual property.

This study utilizes panel data from 280 prefecture-level cities in China spanning from 2007 to 2019, leveraging the Chinese Intellectual Property Demonstration Policy as a quasi-natural experiment to investigate the impact mechanism of IPDP on carbon emission efficiency. The primary research questions addressed in this paper are: can IPDP improve carbon emission efficiency? What are the pathways through which IPDP influences carbon emission efficiency? In addition to its local effects on carbon emission efficiency, can IPDP generate spatial spillover effects on neighboring areas' carbon emission efficiency? The research methodology of this study involves several steps: firstly, a multi-period difference-in-differences model is employed to analyze the impact of IPDP on carbon emission efficiency. The robustness of the findings is confirmed through parallel trend tests, Propensity Score Matching-Difference in Differences (PSM-DID) estimation, exclusion of key cities, exclusion of other policies, placebo tests, and endogeneity tests. Secondly, the sample is stratified based on different city distribution regions, city carbon emissions, and the level of marketization of cities. The cities are categorized into eastern, central, and western regions, cities with higher and lower carbon emissions, and cities with higher and lower levels of marketization to conduct heterogeneity tests, demonstrating the diverse impact of the intellectual property demonstration policy on carbon emission efficiency. Thirdly, a mediation effect model is utilized to examine the indirect impact pathway of the intellectual property demonstration policy on carbon emission efficiency, confirming that the policy influences carbon emission efficiency through green technology innovation and industrial structure upgrading. Lastly, a spatial Durbin difference-in-differences model is applied to assess the spatial spillover effect of the intellectual property demonstration policy on carbon emission efficiency, validating the diffusion effect of the local IPDP on neighboring carbon emission efficiency. Addressing these research questions offers theoretical underpinning and empirical guidance for enhancing the intellectual property protection system, seizing opportunities for institutional innovation, leveraging policy coherence, and promoting green and low-carbon economic development.

The marginal contributions of this paper are threefold: (1) it explores the logical relationship between IPDP and carbon emission efficiency, extending the impact of these policies from the economic to the environmental field, thus enriching the research on the environmental effects of IPDP and providing institutional support and policy pathways for low-carbon green development; (2) it investigates the impact mechanism of IPDP on carbon emission efficiency by examining green technological innovation and industrial structure upgrading, thereby enhancing the theoretical understanding of how intellectual property policies influence carbon emission performance; (3) it assesses the spatial spillover effects of IPDP on carbon emission efficiency in neighboring areas from a spatial correlation perspective, offering new empirical evidence to enhance the radiative effects of demonstration cities, and promoting inter-regional cooperation and coordinated development.

## Related work

### Factors influencing carbon emission efficiency

Various factors have been examined in existing studies to understand the impact of financial development, technological innovation, digital economy, and industrial structure on carbon emission efficiency. In terms of financial development, scholars have debated its effects on carbon emissions. Some argue that financial development exacerbates carbon emissions, with foreign direct investment having a relatively minor impact among financial development indicators^12^. Conversely, others suggest that financial development can reduce carbon emissions by fostering technological innovation and decreasing energy consumption^25^. Regarding technological innovation, Miao et al.^15^ suggest that general technological innovation has a U-shaped effect on carbon emission efficiency, while green technological innovation has a significantly positive impact. Concerning the digital economy, Xie et al.^16^ assert that the digital economy's development notably enhances China's carbon emission efficiency, showing distinct regional variations. Furthermore, Wang et al.^26^ argue that the digital economy can boost the tertiary industry's share and enhance green technological innovation, thereby improving carbon emission efficiency. In terms of industrial structure, Zhou et al.^19^ and Chang et al.^27^ argue that optimizing industrial structure is a viable strategy to enhance carbon emission efficiency. From a policy perspective, prior research has examined the impacts of initiatives like low-carbon city pilots, carbon emission trading rights, and energy use rights trading policies on carbon emission efficiency. Most of these studies suggest that such policies can potentially enhance carbon emission efficiency^20–22^.

### Economic and environmental implications of intellectual property rights protection

The economic implications of intellectual property (IP) protection on technological innovation, foreign investment, and firm productivity have been extensively researched, although the findings are inconclusive. Concerning the influence of IP protection on technological innovation, it is posited that the exclusivity aspect of IP protection can decrease the likelihood of imitation by competitors^28,29^, easing firms' apprehensions about revealing innovative information. This, in turn, allows firms to secure monopoly profits, fostering their inclination towards innovation^30,31^. Conversely, IP protection may impede the advancement of certain small and medium-sized enterprises' leapfrog strategies, potentially diminishing their motivation for innovative endeavors^32,33^. Regarding the impact of IP protection on foreign investment, it is proposed that robust IP protection can impede host countries' ability to replicate the advanced technologies of foreign enterprises, thereby raising the cost of imitation and facilitating the adoption of advanced production technologies from overseas^34–36^. However, excessively stringent IP protection could result in resource wastage and reduced production transfer capabilities, discouraging foreign direct investment^37^. In terms of the effect of IP protection on firm productivity, IP protection can enable firms to license their technologies at premium prices, thereby boosting revenues. It can also alleviate firms from the expenses associated with defending against IP infringements, leading to reduced operational costs and enhanced production efficiency^38^. Nevertheless, knowledge inherently functions as a public good^39,40^, and the knowledge spillover effect can enhance overall industry competitiveness. Consequently, firms are compelled to enhance their productivity to sustain their competitive advantage. Strengthened IP protection may significantly curtail the knowledge spillover effect, impeding productivity enhancements. Moreover, for firms reliant on technology imports, heightened IP protection may drive them towards rent-seeking behaviors, negatively impacting their productivity. Research on the environmental aspect is limited, with only a few scholars exploring the influence of IP protection on environmental pollutants. Di Vita et al.^41^ contend that the IP protection system, as a crucial institutional safeguard for environmental quality, aids in mitigating haze pollution and enhancing environmental conditions.

### Evaluation of the effectiveness of intellectual property demonstration policies

At the corporate level, IPDP has been found to stimulate technological innovation and vitality among enterprises, as evidenced by increased research and development investments, accelerated transformation of scientific and technological achievements, enhanced profitability, improved market pricing power, and promotion of high-quality development. Ultimately, these outcomes enhance market competitiveness and contribute to a rise in total factor productivity^42,43^. On an urban scale, IPDP plays a crucial role in establishing a favorable environment for innovation by reinforcing the enforcement of intellectual property rights, facilitating the rational flow of production factors, and expediting the transfer of production technologies across industries. These efforts support the upgrading of industrial structures within urban areas. Notably, in response to the growing emphasis on energy conservation, emission reduction, and pollution control, IPDP strongly emphasises safeguarding green technologies, thereby elevating the level of green technological innovation in urban settings^44^. Moreover, cities designated as demonstration sites that benefit from IPDP not only receive economic incentives and enhanced prestige but also become more appealing to foreign direct investment, attracting advanced production technologies^45^.

**Figure 1:**
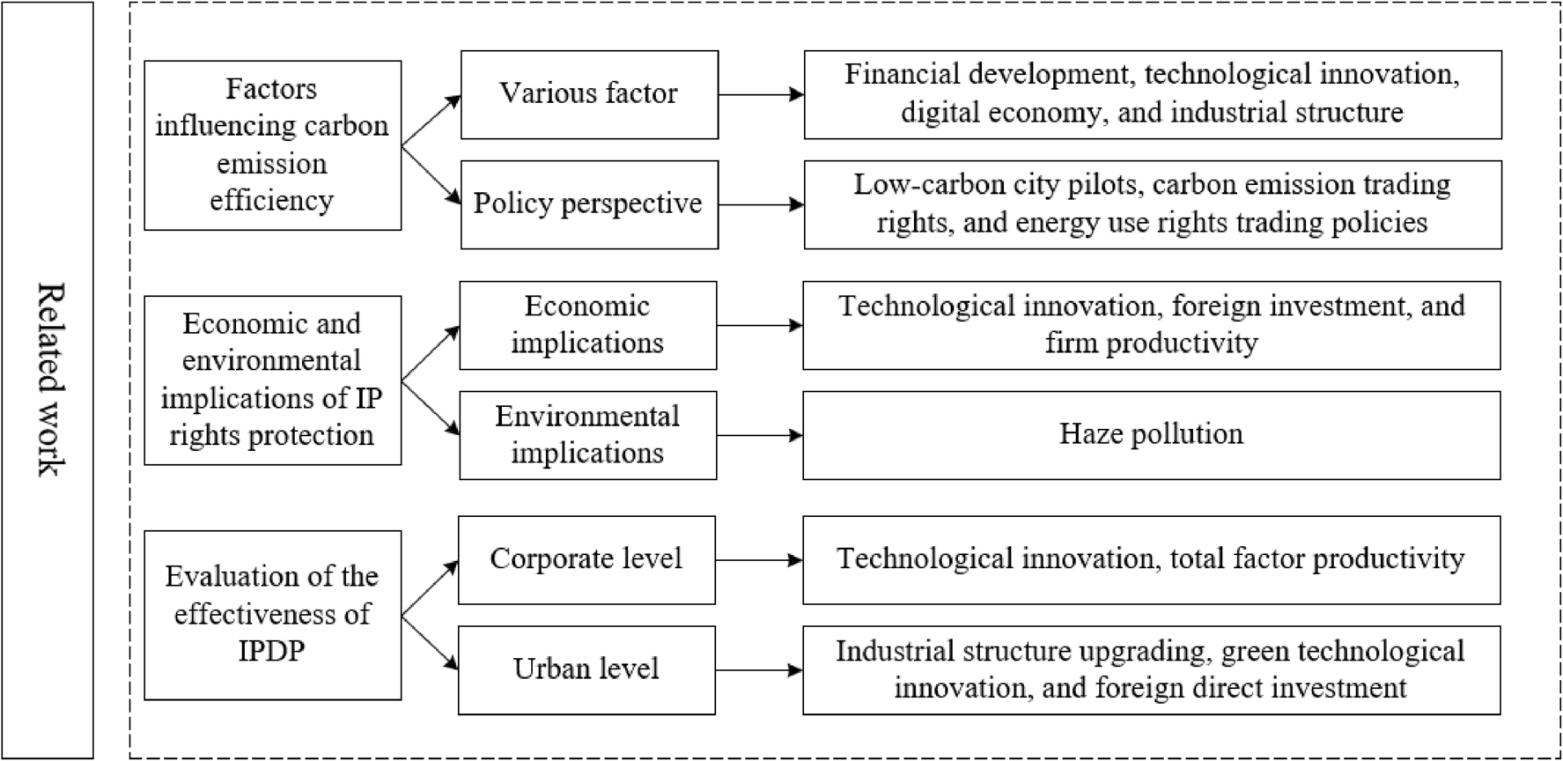
Logical framework of the literature review.

The prevailing literature predominantly concentrates on the economic implications of safeguarding intellectual property, while some researchers delve into its environmental consequences. A few scholars also assess the ramifications of intellectual property demonstration policies within corporate and urban settings, laying a robust groundwork for further exploration in this study. Nevertheless, despite the pivotal role of IPDP in upholding environmental standards, the existing literature lacks comprehensive research on their environmental repercussions and the mechanisms through which they exert influence. To bridge this research gap, this paper endeavors to scrutinize the effects of intellectual property demonstration policies on carbon emission efficiency and the underlying mechanisms. The logical framework of the literature review is depicted in Fig. 1.

## Theoretical analysis and research hypotheses

### Direct impact of IPDP on carbon emission efficiency

The intellectual property demonstration policy facilitates resource allocation optimization, reducing mismatches and the resultant energy consumption, thus lowering carbon emissions and enhancing carbon emission efficiency. Firstly, it optimizes labor allocation. The IPDP has cultivated an urban setting that appreciates knowledge and expertise, supported patent applications, and provided incentives for patent approvals, effectively safeguarding the contributions of highly skilled individuals^46^. This, in turn, accelerates the advancement and implementation of environmentally friendly, low-carbon technologies, thereby improving carbon emission efficiency^47^. Secondly, it streamlines capital allocation. Implementing the IPDP diminishes the risks of patent infringements, encouraging businesses to disclose pertinent information more transparently^48^. Consequently, this helps to reduce information asymmetry between providers and seekers of capital, thereby increasing the likelihood of financial backing^49^. With enhanced access to funding, companies are more motivated to invest in the research and development of green manufacturing technologies and pollution mitigation approaches, thereby bolstering carbon emission efficiency. Lastly, it optimizes technology distribution. The enforcement of the intellectual property demonstration policy underscores protecting intellectual property rights and expediting technology transfer among firms^50,51^. This process attracts sophisticated foreign manufacturing technologies, integrates them into production and pollution control methods, promotes sustainable manufacturing, and improves urban carbon emission efficiency. Based on the insights above, this study posits the following hypothesis.

H1: Intellectual property demonstration policies are conducive to enhancing urban carbon emission efficiency.

### Indirect impact pathways of IPDP on carbon emission efficiency

Green technology innovation plays a crucial role in how IPDP impact the efficiency of carbon emissions. The increasing concerns regarding environmental quality and energy consumption have led to a growing consumer demand for green products. This demand has, in turn, boosted the capabilities of green technological innovation, which is vital for businesses to sustain their competitive advantage in the market^52^. The hesitancy of companies to partake in green innovation activities is mainly attributed to the high costs and risks associated with developing new technologies^53^. Additionally, even successful research and development endeavors can be exposed to significant infringement risks. The integration of IPDP can address these challenges in two key ways: firstly, by establishing supportive funds and innovation subsidies to counteract the issue of inadequate corporate funding, thereby promoting green technology innovation; and secondly, by legally defining the Intellectual Property (IP) rights of businesses in green technologies. This clarification helps reduce the risks and costs linked to technology leakage, minimize the chances of infringements, or provide compensation in cases of infringements. Consequently, this clarification enhances the expected benefits and encourages enterprises to participate in green technology innovation activities^54^.

The advancement of green technology innovations facilitates the shift of production and pollution control technologies towards low-carbon, energy-efficient, and eco-friendly alternatives. This transition helps minimize energy wastage and improve energy efficiency, consequently enhancing carbon emission efficiency. In light of this argument, the following hypothesis is posited:

H2a: Intellectual property demonstration policies enhance urban carbon emission efficiency by improving green technology innovation capabilities.

The upgrading of the industrial structure plays a crucial role in the impact of IPDP on carbon emission efficiency. Firstly, cities designated as demonstration areas benefit from the incentives and recognition associated with IP demonstration policies, which attract a variety of developmental resources. These policies motivate governments to actively compete for additional advantageous resources, creating a solid support system for enhancing industrial structures. Secondly, IPDP creates a favorable environment for intellectual property protection within cities, which appeals to foreign investments and facilitates the introduction of advanced production technologies and well-established management practices to local businesses. This process accelerates the transformation of local industrial structures. Thirdly, implementing IPDP reinforces market mechanisms that support superior enterprises over inferior ones, expediting eliminating high-energy consumption and inefficient businesses. This redirection of resources towards knowledge-intensive industries stimulates the emergence of new business models and industries, ultimately facilitating the upgrading of industrial structures.

Low carbon emission efficiency, often hindered by outdated industrial structures, has the potential for significant enhancement through modernizing these structures. This modernization process gradually replaces less competitive traditional industries with advanced high-tech and high-value-added sectors, thereby reducing environmental degradation and boosting carbon efficiency. Based on this, the following hypothesis is proposed:

H2b: By fostering industrial structural upgrades, intellectual property demonstration policies further enhance urban carbon emission efficiency.

### Spatial spillover effects of IPDP on carbon emission efficiency

Intellectual property demonstration policies not only impact the local carbon emission efficiency but also generate spatial spillover effects on the carbon emission efficiency of adjacent regions. Cities often engage in mutual emulation and learning, fostering healthy competition. In this scenario, IP demonstration cities act as regional models, motivating non-demonstration cities to enhance their IP protection systems, improve governmental IP services, mitigate infringement risks related to green and low-carbon technologies, and promote industrial green transformation, ultimately enhancing carbon emission efficiency. Additionally, implementing IPDP stimulates the establishment of IP conversion and transaction centres^55^, fostering a dynamic technology market. The aggregation, trade, and dissemination of technology create economies of scale, leading to knowledge spillovers and diffusion effects. These mechanisms enable neighboring non-demonstration cities to upgrade their production and pollution control technologies, thereby reducing carbon emissions during production processes and strengthening the capability for end-of-pipe pollution control. Consequently, this process generates a positive spillover effect in enhancing carbon emission efficiency in neighboring cities. Thus, this study proposes hypothesis 3:

H3: Implementing intellectual property demonstration policies has a positive spatial spillover effect on the carbon emission efficiency of adjacent cities, consequently improving their carbon emission efficiency.

## Data and model description

### Data sources

This study employs annual data from 2007 to 2019, collected from 280 prefectural cities in China, to assess the impact of IPDP on carbon emission efficiency. The selection of demonstration cities was based on the list provided by the National Intellectual Property Administration. Information on green patent applications was obtained from the International Patent Classification Green Inventory introduced by the World Intellectual Property Organization in 2010 and the Chinese IP database. Terminology related to environmental protection was extracted from the official reports of each prefecture-level city. Additional research data were obtained from the China City Statistical Yearbook. All price-related variables are adjusted to 2007 levels using appropriate price indices to account for inflation. Descriptive statistics for the variables can be found in Table 1.

**Table 1:** Descriptive statistics for variables.

Variable	Observation	Mean	Standard deviation	Minimum	Maximum
CEE	3640	0.543	0.259	0.073	2.442
PDV	3640	0.211	0.408	0.000	1.000
TDV	3640	0.098	0.297	0.000	1.000
GTIC	3640	1.150	2.875	0.000	49.982
ISU	3640	0.399	0.103	0.086	0.835
EDL	3640	1.058	0.915	0.075	20.100
ER	3640	0.003	0.001	0.000	0.012
ES	3640	0.651	0.176	0.000	1.000
PSI	3640	0.477	0.110	0.019	0.910

CEE: Carbon Emission Efficiency; PDV: Policy Dummy Variable; TDV: Time Dummy Variable; GTIC: Green Technological Innovation Capability; ISU: Industrial Structure Upgrade; EDL: Economic Development Level; ER: Environmental Regulation; ES: Energy Structure; PSI: Proportion of Secondary Industry.

### Model design

In the contemporary era of ecological civilization, the significance of green development is paramount, necessitating the essential support of intellectual property law for green and low-carbon technologies. The intellectual property system serves functions such as substitution, guidance, incentive, and regulation, which are beneficial for promoting ecological technology transformation, recycling resources, and improving carbon emission efficiency across society. To effectively assess the impact of IPDP on carbon emission efficiency, this study utilizes these policies as a quasi-natural experiment, classifying pilot and non-pilot cities as treatment and control groups, and constructs a difference in differences (DID) model to assess the policy implementation outcomes. Unlike traditional DID models that assume policy implementation co-occurs, this analysis recognizes that IP demonstration cities were designated in successive phases over different years. Consequently, this study built the following multiple-time-point DID model^56^:

1 $$CEE_i,_t = \beta_0 + \beta_1 \times IPDP_{i,t} + \theta_1 \times X_{i,t} + \varepsilon_{i,t}$$

where $CEE$ denotes the carbon emission efficiency, $\beta 0$, $\beta 1$ and $\theta 1$ represent estimated coefficients within the statistical model, $X$ encompasses the control variables, and $\varepsilon$ signifies the stochastic error term, with the subscripts $i$ and $t$ identifying city and year, respectively.

Furthermore, to elucidate the transmission mechanism by which the IPDP impacts carbon emission efficiency, we have developed models (2) and (3). These models are integrated with the previously mentioned model (1) to compose a mediation effect framework, enabling a thorough analysis of the policy's influence.

2 $$M_{i,t} = \alpha_0 + \alpha_1 \times IPDP_{i,t} + \theta_2 \times X_{i,t} + \varepsilon_{i,t}$$

3 $$CEE_{i,t} = \gamma_0 + \gamma_1 \times IPDP_{i,t} + \gamma_2 \times M_{i,t} + \theta_3 \times X_{i,t} + \varepsilon_{i,t}$$

where $M$ denotes the mediating variables, specifically the capability for green technological innovation and upgrading industrial structures, $\alpha 0$, $\alpha 1$, $\gamma 0$, $\gamma 1$, $\gamma 2$, $\theta 2$ and $\theta 3$ represent estimated coefficients within the statistical model.

### Variable descriptions

(1) Dependent variable.

Carbon emission efficiency (CEE): we employ the non-desired output Super-SBM model to evaluate the CEE of Chinese prefecture-level cities, overcoming the limitation of traditional SBM models that cannot rank multiple decision-making units with efficiency scores of 1. The evaluation of CEE considers various input factors, including capital, labor, and energy. Capital input is determined by the fixed capital stock, estimated using the perpetual inventory method with 2007 as the base year and adjusted for price using the provincial fixed asset investment indices. Labor input is measured by the total number of employees at year-end in each city, while the total annual electricity consumption in each city quantifies energy input. The assessment of CEE differentiates between desired and undesired outputs. The desired output is the real Gross Domestic Product (GDP), calculated by adjusting nominal GDP using the provincial GDP price index with 2007 as the base year. The undesired output comprises total carbon emissions, which include emissions within the city, those associated with energy consumption outside the jurisdiction, and emissions resulting from urban activities in the town but occurring beyond its boundaries.

(2) Core explanatory variable.

Intellectual Property Demonstration Policy (IPDP): the proxy variable for the IPDP is the interaction term between the policy dummy variable (PDV) and the time dummy variable (TDV). The PDV distinguishes whether a prefecture-level city has been designated as an IP demonstration city, assigning a value of 1 to cities under the designation and 0 to others. The TDV signifies the timeframe during which the IPDP is active; it is assigned a value of 1 during and after the policy's implementation in a city, and 0 for years preceding implementation or in cities that have not yet adopted the policy.

(3) Mediating variable.

Green Technological Innovation Capability (GTIC): green patents are the direct output of enterprises engaging in green technological innovation activities, reflecting the enterprises' capabilities in low-carbon production and pollution management. The quantity of green patent applications per ten thousand individuals within a city serves as a gauge of the city's prowess in green technological innovation. This study employs the number of green patent applications per ten thousand people as a surrogate indicator for assessing green innovation capability.

Industrial Structure Upgrade (ISU): the upgrade primarily refers to transforming and developing traditional and emerging new industries. This study uses the proportion of added value in the tertiary industry as a proxy indicator for upgrading the industrial structure.

(4) Control variables.

Economic Development Level (EDL): Initially, an increase in the economic development level may result in a deterioration of environmental quality. However, as the economic development level progresses, businesses are more likely to adopt cleaner production technologies, improving environmental quality. The natural logarithm of real per capita GDP is used as the measurement indicator, and a quadratic term of economic development level (EDL^2^) is included to examine the conformity of the relationship between the level of economic development and carbon emission efficiency with the Environmental Kuznets Curve hypothesis.

Environmental Regulation (ER): Stringent environmental regulations have the potential to drive technological innovation within enterprises, leading to enhanced energy utilization efficiency and improved carbon emission efficiency. Conversely, these regulations may also escalate pollution control expenses for enterprises, diverting financial resources away from technological advancements and impeding progress in carbon emission efficiency. The metric used to gauge this phenomenon is the proportion of words related to environmental protection in the municipal government reports to the total word count of these reports.

Energy Structure (ES): Energy structures characterized by high energy consumption and pollution-intensive enterprises typically rely heavily on coal. The low-carbon transformation of the energy structure contributes to enhancing carbon emission efficiency. The chosen measurement indicator is the proportion of industrial electricity consumption to total social electricity consumption in society.

Proportion of Secondary Industry (PSI): A more significant share of the secondary industry may impede the enhancement of carbon emission efficiency. The proportion of the value added by the secondary industry to the GDP is utilized as the metric for assessment.

## Empirical results and analysis

**Figure 2:**
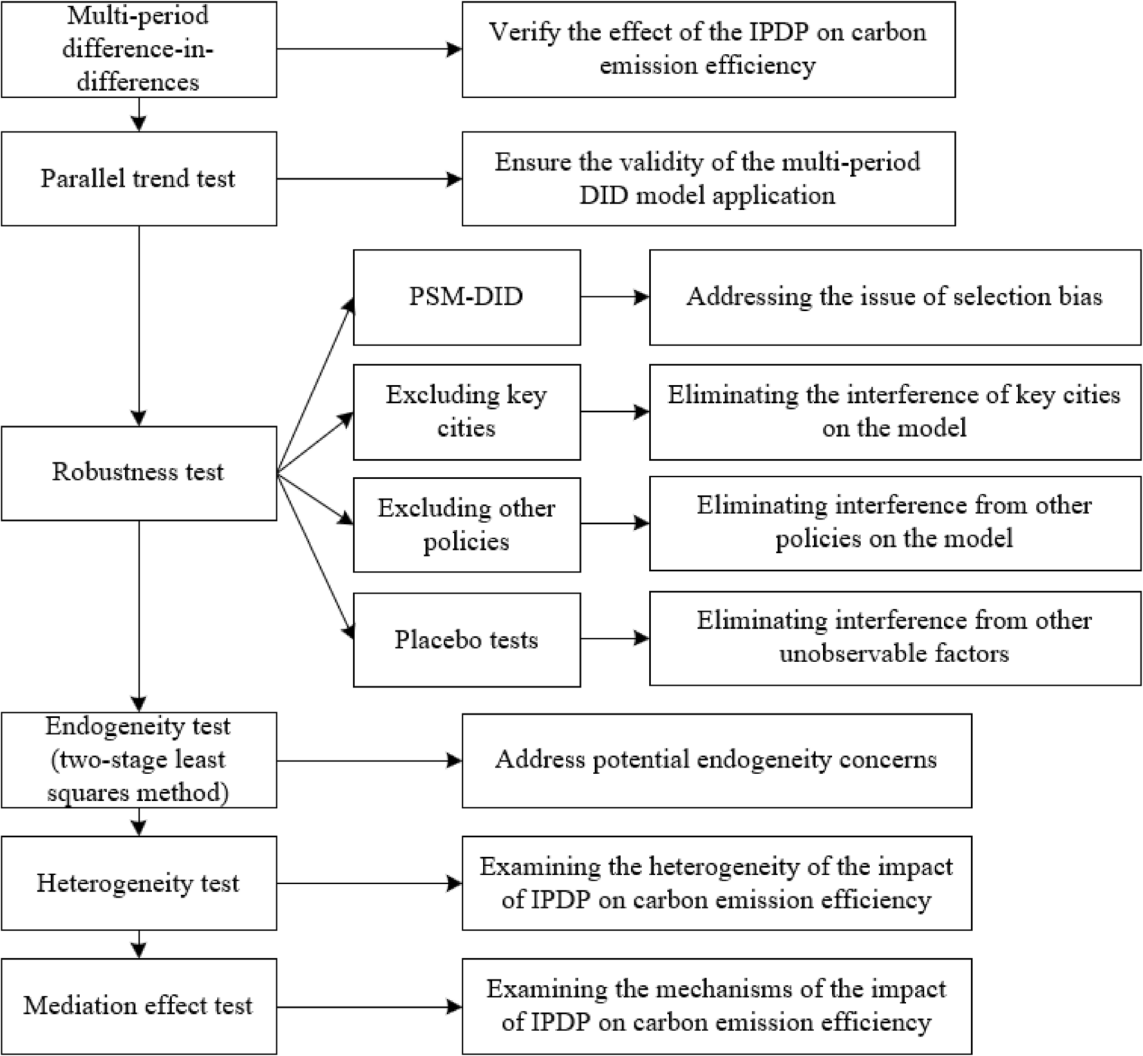
A framework of empirical analysis methods.

We conducted a series of empirical analyses to comprehensively understand the impact of the IPDP on carbon emission efficiency. Firstly, the IPDP was employed as a quasi-natural experiment to assess its effects, with a multi-period Difference-in-Differences (DID) model utilized for baseline regression. A positive and statistically significant regression coefficient for the IPDP suggests that it can improve carbon emission efficiency. Secondly, we employed the parallel trend method to assess the suitability of the multi-period DID model by examining whether there were significant differences in carbon emission efficiency between demonstration and non-demonstration cities before the policy implementation. Meeting this criterion would validate the appropriateness of our multi-period DID model. Thirdly, to ensure the reliability of our baseline model findings, we conducted various robustness checks, including Propensity Score Matching-DID (PSM-DID), exclusion of critical cities, exclusion of other policies, and placebo tests. The PSM-DID method was used to address selection bias, while the exclusion of crucial city controls aimed to mitigate potential interference, and the exclusion of other policy controls accounted for the effects of policies such as "Smart City" and "Broadband China." Additionally, placebo tests were conducted to eliminate the influence of other unobservable factors. If the regression coefficient of the IPDP remains significantly positive following these tests, it would confirm the robustness of the conclusion that the IPDP enhances carbon emission efficiency. Fourth, considering potential endogeneity issues in the baseline model, the number of Confucian academies in different prefecture-level cities in China was selected as an instrumental variable for the IPDP, and the Two-Stage Least Squares method was employed for endogeneity testing. If the regression coefficient of the IPDP remains significantly positive in the endogeneity test, it further supports the robustness of the conclusion that the IPDP promotes carbon emission efficiency. Fifth, acknowledging that the impact of the IPDP on carbon emission efficiency may differ across regions, carbon emission levels, and degrees of marketization, the total sample was divided into various subgroups for heterogeneity testing. By comparing the magnitude and significance of the regression coefficients of the IPDP across different samples, the varying impacts of the IPDP on carbon emission efficiency in cities with different characteristics were determined. Sixth, a mediation effect model was constructed to validate the mechanisms through which the IPDP influences carbon emission efficiency, utilizing green technological innovation and industrial structure upgrading as mediating variables to confirm the transmission paths of these factors. These empirical tests confirmed the impact and mechanisms of the IPDP on carbon emission efficiency, expanding the scope of the IPDP's influence from economic to environmental realms. This study enriches research on the environmental efficacy of the IPDP and enhances the theoretical understanding of factors affecting carbon emission efficiency. The research methodologies employed in this study are depicted in Fig. 2.

### Empirical results of the baseline regression

Table 2 displays the baseline regression results, gradually adding control variables in columns (1)–(6). The results reveal significantly positive regression coefficients for the IPDP in all columns. For example, in column (6), the coefficient for IPDP is 0.044, indicating that a 1% increase in policy implementation boosts carbon emission efficiency by 0.044%. The study suggests that IPDP promotes a city environment that values knowledge and talent, reduces patent infringement risks for businesses, introduces advanced foreign technologies, enhances information disclosure, and improves financing prospects. Moreover, the policy attracts top talent, technologies, and capital, leading to production and pollution control technology advancements, ultimately enhancing carbon emission efficiency. These findings support Hypothesis 1, as Pan et al.^57^ confirmed in their study on the IPDP's impact on carbon emission efficiency.

**Table 2:** Empirical results of baseline regression.

Variable	(1)	(2)	(3)	(4)	(5)	(6)
IPDP	0.038*** (0.014)	0.055*** (0.014)	0.064*** (0.014)	0.065*** (0.014)	0.055*** (0.014)	0.044*** (0.014)
EDL		− 0.042*** (0.004)	− 0.077*** (0.006)	− 0.079*** (0.006)	− 0.080*** (0.006)	− 0.092*** (0.007)
EDL^2^			0.004*** (0.001)	0.004*** (0.001)	0.004*** (0.001)	0.005*** (0.001)
ER				5.792** (2.489)	5.067** (2.472)	5.057** (2.466)
ES					− 0.235*** (0.032)	− 0.226*** (0.032)
PSI						− 0.238*** (0.057)
Constant	0.539*** (0.003)	0.582*** (0.005)	0.610*** (0.007)	0.593*** (0.010)	0.751*** (0.024)	0.871*** (0.038)
Observations	3640	3640	3640	3640	3640	3640

IPDP: Intellectual Property Demonstration Policies; EDL: Economic Development Level; EDL^2^: a quadratic term of economic development level; ER: Environmental Regulation; ES: Energy Structure; PSI: Proportion of Secondary Industry. The symbols *, **, and *** denote statistical significance at the 10%, 5%, and 1% levels, respectively, while the values in parentheses indicate standard errors.

The regression results for controlled variables indicate that the coefficient for the primary term of EDL is significantly negative, whereas the coefficient for EDL^2^ is significantly positive, demonstrating an inverted-U relationship between economic development and carbon emission efficiency. This finding is consistent with the Environmental Kuznets Curve hypothesis, which posits that environmental pollution worsens initially with economic growth but improves once a certain economic threshold is reached. Additionally, the positive regression coefficient for ER suggests that stringent environmental regulations prompt firms to adopt energy-efficient production methods and enhance pollution control technologies, thereby improving carbon emission efficiency^58^. Conversely, the negative coefficient for ES implies that a higher proportion of energy consumption harms environmental quality and hinders improving carbon emission efficiency. In particular, the significantly negative coefficient for PSI indicates that a higher industrial share, reflecting a greater dependence on the industrial sector, is unfavorable for enhancing carbon emission efficiency.

### Empirical results of the parallel trend test

The prerequisite for applying a multiple-time-point DID model is to verify the existence of a parallel trend in carbon emission efficiency between the treatment and control group cities before implementing the IPDP. Thus, we construct time dummy variables for the two years before and the year of policy implementation, setting up the following regression model to test for parallel trends^59^:

4 $$CEE_{i,t} = \gamma_{0} + \gamma_{k} \sum\nolimits_{\delta =0}^{{\delta { = } - 2}} {\left( {IPDP_{i,t} \times year\_\delta } \right)} + \gamma_{1} X_{i,t} + \varepsilon_{i,t}$$

where $\gamma 0$ and $\gamma 1$ represent estimated coefficients within the statistical model, $\gamma_{k}$ denotes the regression coefficient, $year\_\delta$ represents the time dummy variable for the two years prior to and including the year of IPDP implementation. The IPDP was rolled out in six batches, with $year\_{- 2}$ corresponding to 2010, $year\_- 1$ to 2011, $year\_{0}$ to 2012 for the first batch, and similarly for subsequent batches. We conduct batch-specific and sample-specific tests to mitigate the interference between different batches.

Table 3 presents the outcomes of the parallel trends test. The results show that out of the six batches examined, solely the regression coefficient for $year\_0$ in the 2013 batch exhibited a statistically significant positive value. In contrast, the other regression coefficients were statistically non-significant, suggesting a lack of notable distinction in carbon emission efficiency between the experimental and control group cities before implementation of the IPDP. Consequently, this observation confirms the fulfillment of the parallel trend assumption.

**Table 3:** Empirical results of the parallel trend test.

Variable	Batch 2012	Batch 2013	Batch 2015	Batch 2016	Batch 2018	Batch 2019
Year 2010	− 0.031 (0.060)					
Year 2011	− 0.027 (0.048)	− 0.017 (0.047)				
Year 2012	− 0.011 (0.047)	− 0.002 (0.047)				
Year 2013		0.064* (0.036)	0.019 (0.030)			
Year 2014			− 0.022 (0.029)	− 0.027 (0.029)		
Year 2015			− 0.035 (0.029)	− 0.040 (0.029)		
Year 2016				− 0.013 (0.029)	− 0.001 (0.029)	
Year 2017					0.005 (0.028)	0.009 (0.028)
Year 2018					0.042 (0.028)	0.046 (0.028)
Year 2019						0.042 (0.029)
Control variable	Yes	Yes	Yes	Yes	Yes	Yes
Constant	1.847*** (0.154)	1.860*** (0.154)	1.831*** (0.156)	1.811*** (0.157)	1.838*** (0.156)	1.836*** (0.154)
Observations	3640	3640	3640	3640	3640	3640

The symbols *, **, and *** denote statistical significance at the 10%, 5%, and 1% levels, respectively, while the values in parentheses indicate standard errors.

### Empirical results of the robustness analysis

(1) Propensity score matching DID estimation.

Potential selection bias may arise due to the non-random selection of IP demonstration cities. We employ the Propensity Score Matching Difference in Differences (PSM-DID) method to mitigate this issue as a robustness check. To enhance the reliability of the matching results, we utilize three different matching techniques: 1:1 nearest neighbor matching, radius matching, and kernel matching. These techniques are applied in regressions with four control variables (i.e., EDL, ER, ES, PSI) as matching variables to develop a logit model that determines the designation of a city as an IP demonstration city. Each matching technique is employed to create a matched sample, which is re-evaluated using the DID model.

**Figure 3:**
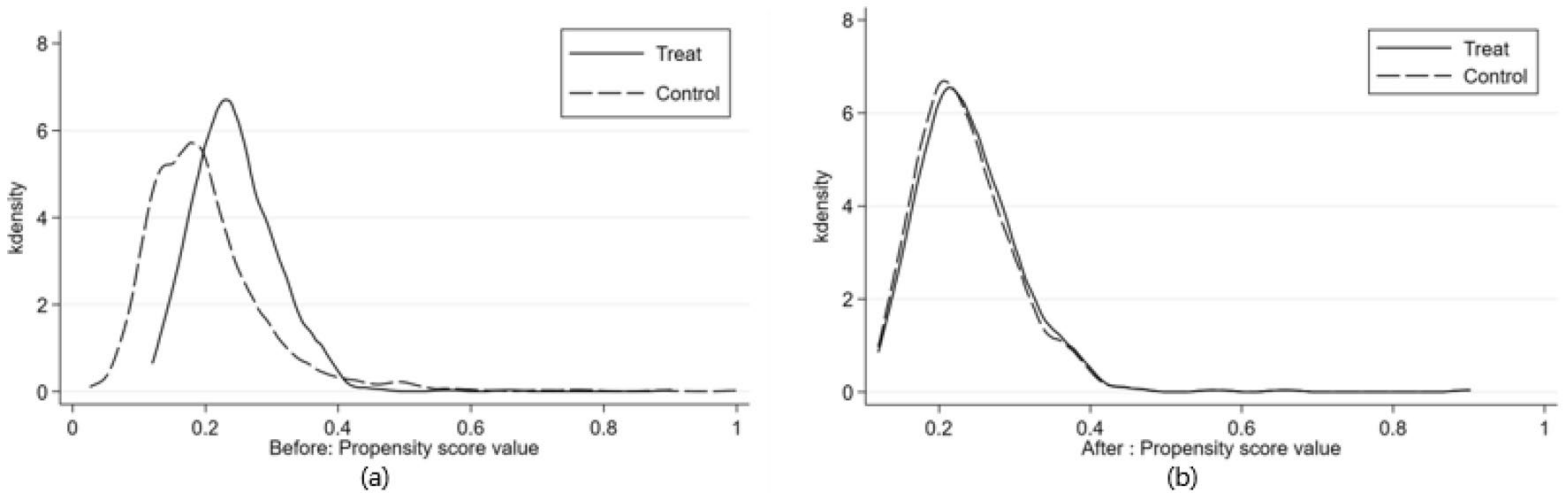
The kernel density plots of propensity scores; (**a**) before matching; (**b**) after matching.

Before re-running the regressions with the matched samples, Fig. 3 displays kernel density plots illustrating the propensity score for the experimental and control groups through 1:1 nearest neighbor matching. Figure 3a reveals a substantial disparity in the propensity score distributions between the treatment and control groups before matching, with the control group exhibiting notably lower scores, suggesting evident sample selection bias. Following the matching process, Fig. 3b showcases the propensity score distributions of the treatment and control groups converging closely, indicating no substantial variances and meeting the common support assumption necessary for propensity score matching.

Table 4 presents the results of the regression analyses conducted with the DID model on the propensity score-matched samples, where columns (1) through (3) correspond to distinct matching techniques. The results indicate that the coefficients related to the IPDP exhibit a statistically significant positive association, consistent with the baseline regression results, thus reaffirming the research hypothesis that the IPDP contributes to improving carbon emission efficiency.

**Table 4:** Empirical results of the robustness.

Variable	Propensity score matching			Exclude key cities	Exclude the influence of other policies	
	Nearest neighbor matching	Radius matching	Kernel matching			
	(1)	(2)	(3)	(4)	(5)	(6)
IPDP	0.048** (0.019)	0.043*** (0.014)	0.043*** (0.014)	0.030** (0.014)	0.035** (0.014)	0.034** (0.015)
EDL	− 0.134*** (0.027)	− 0.103*** (0.014)	− 0.102*** (0.014)	− 0.094*** (0.007)	− 0.097*** (0.007)	− 0.095*** (0.007)
EDL^2^	0.005 (0.004)	0.005** (0.002)	0.005** (0.002)	0.005*** (0.001)	0.005*** (0.001)	0.005*** (0.001)
ER	− 5.039 (5.128)	4.438 (2.700)	4.577* (2.616)	4.743* (2.429)	3.937 (2.472)	4.422* (2.471)
ES	− 0.217*** (0.077)	− 0.212*** (0.036)	− 0.209*** (0.035)	− 0.216*** (0.032)	− 0.213*** (0.032)	− 0.220*** (0.032)
PSI	− 0.555*** (0.164)	− 0.319*** (0.072)	− 0.312*** (0.069)	− 0.211*** (0.057)	− 0.213*** (0.058)	− 0.213*** (0.059)
Smart Cities					0.042*** (0.011)	
Broadband China						0.023** (0.011)
Constant	1.170*** (0.110)	0.927*** (0.048)	0.917*** (0.046)	0.849*** (0.037)	0.853*** (0.038)	0.858*** (0.038)
Observations	1187	3208	3293	3640	3640	3640

IPDP: Intellectual Property Demonstration Policies; EDL: Economic Development Level; EDL^2^: a quadratic term of economic development level; ER: Environmental Regulation; ES: Energy Structure; PSI: Proportion of Secondary Industry. The symbols *, **, and *** denote statistical significance at the 10%, 5%, and 1% levels, respectively, while the values in parentheses indicate standard errors.

(2) Analysis excluding key cities.

Since directly administered municipalities and major first-tier cities tend to possess a heightened awareness of environmental conservation and employ more advanced production and pollution control technologies, their involvement could skew the evaluation of the impacts of IPDP. Therefore, this research excludes Beijing, Tianjin, Shanghai, Chongqing, Guangzhou, and Shenzhen from the sample to mitigate this bias. The analysis then focuses on the remaining cities, as detailed in Column (4) of Table 4. The findings of the study demonstrate a notably positive regression coefficient associated with the IPDP, affirming the robustness of the baseline results and indicating that the IPDP enhances carbon emission efficiency across all pilot cities, not just a select few.

(3) Analysis excluding the influence of other policies.

This study also accounts for the impact of additional pilot policies implemented during the sample period that may influence carbon emission efficiency. Previous studies have suggested that implementing the Smart Cities and Broadband China pilot programs can improve carbon emission efficiency. Thus, we incorporate dummy variables for these two policies into the baseline regression model. As the implementation of the Smart Cities and Broadband China policies occurred in phases, we assigned a value of 0 for the period before and 1 for the implementation year and subsequent years. The regression results from columns (5) and (6) of Table 4 show a significantly positive coefficient for the IPDP, indicating that even after controlling for the effects of both policy types, the IPDP continues to enhance carbon emission efficiency.

(4) Placebo test.

**Figure 4:**
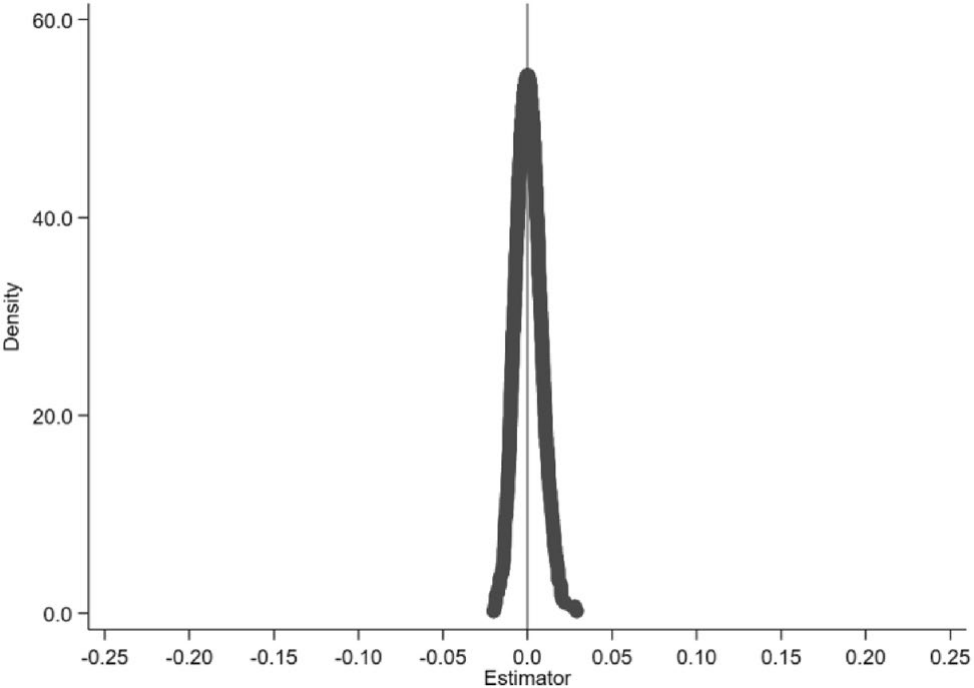
Results of a placebo test conducted 1000 times through sampling.

Considering that unobservable factors might influence the baseline regression outcomes, this study incorporates a placebo test to validate the robustness of the baseline model's regression results^60^. Specifically, we randomly selected 59 cities from the total sample (corresponding to the number of cities in the experimental group in the baseline regression) to establish a pseudo-experimental group, assuming that these cities represent intellectual property demonstration cities, while the remaining cities were designated as the pseudo-control group. Subsequently, using Model (1), we analyzed these cities through regression testing. To mitigate the randomness of the pseudo-experimental group selection, this procedure is iterated 1,000 times, and the probability density distribution of the IPDP regression coefficients is observed, as shown in Fig. 4. The regression coefficient of IPDP tends towards zero, and its distribution is highly similar to a normal distribution, indicating that other unobservable factors do not influence the baseline regression results.

### Empirical results of the endogeneity check

The multiple-time-point DID model partially addresses endogeneity issues. Nevertheless, the non-random selection process of IPDP and the tendency to choose cities with higher carbon emission efficiency due to their advanced production and pollution control technologies introduce bidirectional causality, leading to additional endogeneity concerns. To mitigate this issue, this study employs the instrumental variable method, utilizing the number of Confucian Academies (CA) in each prefecture-level city as instrumental variables for IPDP. The data is obtained from the Confucian Culture Database, available on the Chinese Research Data Services Platform. The CA serves as a relevant instrumental variable as regions with many CAs are more likely to be strongly aware of intellectual property rights, increasing their chances of being designated IP demonstration cities. Furthermore, the CA meets the homogeneity condition as ancient academies in cities are only weakly associated with carbon emission efficiency.

Table 5 presents the regression outcomes obtained through the two-stage least squares technique. The first-stage regression analysis demonstrates an F-value exceeding 10, signifying the absence of weak instrumental variables. Additionally, the positive and statistically significant coefficient for CA implies that a greater number of CAs heightens the probability of a city's selection as an IP demonstration city. In the second-stage regression analysis, the positive and significant coefficient for IPDP persists, even after mitigating endogeneity concerns, indicating that the IPDP consistently improves carbon emission efficiency. Consequently, the baseline regression results remain unaffected by endogeneity challenges.

**Table 5:** Empirical results of endogeneity check.

Variable	First-stage regression (IPDP)	Second-stage regression (CEE)
CA	0.003*** (0.001)	
IPDP		1.362*** (0.258)
EDL	0.114*** (0.009)	− 0.203*** (0.033)
EDL^2^	− 0.008*** (0.001)	1.012*** (0.002)
ER	− 3.677 (3.389)	6.668 (4.931)
ES	− 0.047 (0.031)	− 0.032 (0.047)
PSI	0.200*** (0.052)	− 0.127 (0.093)
Constant	0.082*** (0.029)	0.660*** (0.046)
Observations	3640	3640
First-stage F-value	31.88	

CA: Confucian Academies; IPDP: Intellectual Property Demonstration Policies; CEE: Carbon emission efficiency; EDL: Economic Development Level; EDL^2^: a quadratic term of economic development level; ER: Environmental Regulation; ES: Energy Structure; PSI: Proportion of Secondary Industry. The symbols *, **, and *** denote statistical significance at the 10%, 5%, and 1% levels, respectively, while the values in parentheses indicate standard errors.

### Empirical results of the heterogeneity test

Baseline regression results suggest that the IPDP significantly improves carbon emission efficiency, although its impact varies depending on the urban distribution areas and specific city characteristics. Thus, this study conducts the following heterogeneity tests:

(1) Heterogeneity of urban distribution areas. Considering the significant disparities in economic development levels and infrastructure construction between cities in the eastern region and the central and western regions, the impact of IPDP on carbon emission efficiency may differ across these areas. This study divides the sample cities based on their geographical location into eastern regions and west and central regions for heterogeneity testing, as presented in Table 6. The experimental results reveal that the regression coefficient of IPDP is significantly positive in eastern region cities, while it is not statistically significant in central and western region cities, suggesting that IPDP positively influences carbon emission efficiency only in cities located in the eastern region. The underlying reason is that the natural geographical advantages and higher economic development level of the east region, which foster a business environment more attuned to IP protection. Consequently, implementing IPDP in these cities promotes corporate innovation, facilitates industrial restructuring, and improves carbon emission efficiency. In contrast, the central and western regions, characterized by lower economic development levels and limited initial resources such as talent and capital, face challenges in achieving rapid transformation and upgrades, thereby reducing the effectiveness of IPDP in enhancing carbon emission efficiency.

**Table 6:** Empirical results of heterogeneity check.

Variable	Heterogeneity of urban distribution areas		Heterogeneity of urban carbon emissions		Heterogeneity in the degree of marketization across cities	
	Easterncities	Midwest cities	Cities with lower carbon emissions	Cities with higher carbon emissions	Cities with a low degree of marketization	Cities with a higher degree of marketization
IPDP	0.074*** (0.021)	0.030 (0.019)	0.052*** (0.020)	0.034* (0.020)	0.017 (0.019)	0.068*** (0.021)
EDL	− 0.122*** (0.023)	− 0.077*** (0.007)	− 0.081*** (0.008)	− 0.104*** (0.012)	− 0.094*** (0.010)	− 0.087*** (0.010)
EDL^2^	0.006 (0.004)	0.004*** (0.001)	0.005*** (0.001)	0.005*** (0.001)	0.005*** (0.001)	0.004*** (0.001)
ER	2.715 (4.817)	6.819** (2.737)	5.997** (3.051)	4.904 (3.923)	− 0.786 (3.406)	10.490*** (3.555)
ES	− 0.152** (0.069)	− 0.209*** (0.033)	− 0.124*** (0.039)	− 0.349*** (0.053)	− 0.252*** (0.047)	− 0.208*** (0.045)
PSI	− 0.634*** (0.131)	− 0.029 (0.058)	− 0.050 (0.069)	− 0.438*** (0.097)	− 0.164** (0.076)	− 0.332*** (0.089)
Constant	1.159*** (0.084)	0.679*** (0.038)	0.653*** (0.047)	1.100*** (0.060)	0.863*** (0.051)	0.891*** (0.056)
Observations	1300	2340	1820	1820	1820	1820

IPDP: Intellectual Property Demonstration Policies; EDL: Economic Development Level; EDL^2^: a quadratic term of economic development level; ER: Environmental Regulation; ES: Energy Structure; PSI: Proportion of Secondary Industry. The symbols *, **, and *** denote statistical significance at the 10%, 5%, and 1% levels, respectively, while the values in parentheses indicate standard errors.

(2) Heterogeneity of urban carbon emissions. The IPDP serves as a crucial institutional mechanism to enhance environmental quality. Due to notable disparities in carbon emissions levels across different cities, the influence of IPDP on carbon emission efficiency may vary depending on the emissions levels of the cities. To assess this, we aggregated carbon emissions within each prefectural city and emissions related to energy consumption and urban activities outside the city's jurisdiction to determine the total carbon emissions for each city. Subsequently, we categorized the sampled cities into those with lower and higher carbon emissions based on the average total emissions, and conducted heterogeneity testing, as detailed in Table 6. The findings reveal that the regression coefficients of IPDP are significantly positive in cities with low and high carbon emissions. However, the coefficient in cities with lower emissions (0.052) surpasses that in cities with higher emissions (0.034), indicating that IPDP has a more pronounced impact on improving carbon emission efficiency in cities with lower emissions. This discrepancy can be attributed to cities with higher carbon emissions often grappling with severe environmental pollution and predominantly relying on primary and secondary industries, which may lack inherent innovation capabilities and frequently resort to advanced pollution control and carbon reduction technologies. Nevertheless, implementing IPDP has increased costs associated with technology transfer, resulting in a crowding-out effect that hampers enhancing carbon emission efficiency in these cities.

(3) Heterogeneity in the degree of marketization across cities. The IPDP can incentivize enterprises to adopt green technological innovations. Promoting green technologies can aid in the rational distribution of resources among enterprises, thereby enhancing carbon emission efficiency. Nevertheless, the level of marketization varies among cities. Cities with higher levels of marketization experience benefits such as streamlined governance and decentralization, which facilitate optimal resource allocation. This results in differing impacts of IPDP on carbon emission efficiency across cities with varying levels of marketization. We use the proportion of employees in private and individual enterprises to total employment to calculate the degree of marketization in each city. Based on the average marketization level of the sample cities, the total sample is divided into cities with lower and higher degrees of marketization for heterogeneity testing, as shown in Table 6. The results indicate that in cities with higher degrees of marketization, the regression coefficient of IPDP is significantly positive. In contrast, the coefficient is insignificant in cities with lower marketization levels, suggesting that IPDP enhances carbon emission efficiency predominantly in cities with advanced market mechanisms. The underlying cause is that in cities with lower levels of marketization, extensive governmental intervention in markets leads to misallocation and wastage of resources, ultimately hampers the improvement of carbon emission efficiency.

### Empirical results of the mechanism check

The baseline regression results suggest that the IPDP enhances carbon emission efficiency. To elucidate the underlying mechanisms, this study examines the effects of mediation on verifying hypotheses 2a and 2b.

The baseline regression results indicate that IPDP enhances carbon emission efficiency. What is the transmission mechanism behind this? Based on this, the present study conducts a test of the mediating effect model to examine the mechanism of action and verify hypotheses 2a and 2b.

(1) Mechanism check for green technological innovation. Column 3 of Table 7 displays the regression outcomes concerning the influence of IPDP on green technological innovation capabilities. The coefficient associated with the IPDP is significantly positive, suggesting that IPDP can stimulate firms to participate in green technological innovation endeavors and improve their abilities. In Column 4, green technological innovation capabilities are integrated into Model (1) for regression examination. Despite the continued positive significance of the IPDP, the coefficient (0.029) in Column 2 of Table 7 is noted to be lower than its previous value (0.044). The adoption of the IPDP has the potential to mitigate the financial constraints encountered by enterprises involved in green technological innovation, decrease the risk costs linked to technology loss, and enhance the anticipated returns from activities related to green technological innovation. Consequently, this policy plays a role in fostering the enhancement of urban green technological innovation capacity, leading to advancements in energy efficiency and increased carbon emission efficiency^61^.

**Table 7:** Empirical results of mechanism check.

Variable	Model (1)	Model (2)	Model (3)	Model (2)	Model (3)
	CEE	GTIC	CEE	ISU	CEE
IPDP	0.044*** (0.014)	3.692*** (0.129)	0.029* (0.015)	0.016*** (0.002)	0.034** (0.014)
GTIC			0.012*** (0.002)		
ISU					0.593*** (0.118)
EDL	− 0.092*** (0.007)	0.469*** (0.066)	− 0.093*** (0.007)	0.048*** (0.001)	− 0.120*** (0.009)
EDL^2^	0.005*** (0.001)	− 0.027*** (0.005)	0.005*** (0.001)	− 0.002*** (0.000)	0.006*** (0.001)
ER	5.057** (2.466)	− 82.385*** (22.971)	5.669** (2.459)	2.376*** (0.358)	3.648 (2.473)
ES	− 0.226*** (0.032)	− 0.542* (0.306)	− 0.189*** (0.030)	− 0.028*** (0.005)	− 0.209*** (0.032)
PSI	− 0.238*** (0.057)	− 4.260*** (0.535)	− 0.082 (0.053)	− 0.735*** (0.008)	0.198* (0.104)
Constant	0.871*** (0.038)	3.008*** (0.352)	0.762*** (0.035)	0.711*** (0.005)	0.449*** (0.092)
Observations	3640	3640	3640	3640	3640

IPDP: Intellectual Property Demonstration Policies; CEE: Carbon emission efficiency; GTIC: Green Technological Innovation Capability; ISU: Industrial Structure Upgrade; EDL: Economic Development Level; EDL^2^: a quadratic term of economic development level; ER: Environmental Regulation; ES: Energy Structure; PSI: Proportion of Secondary Industry. The symbols *, **, and *** denote statistical significance at the 10%, 5%, and 1% levels, respectively, while the values in parentheses indicate standard errors.

(2) Mechanism check for industrial structure upgrading. Column 5 of Table 7 presents the regression results regarding the influence of IPDP on upgrading industrial structures. The results reveal a significantly positive coefficient, implying that IPDP supports introducing novel business models and optimizing industrial structure. Subsequently, after incorporating industrial structure enhancement into Model (1), as illustrated in Column 6, IPDP retains a positive coefficient. However, the magnitude of the coefficient (0.034) is slightly lower than that in Column 2 (0.044), suggesting that IPDP assists demonstration cities in attracting more favorable resources and foreign investments. This, in turn, reinforces the competitive selection mechanism in the market and fosters emerging industries. Consequently, there is a gradual phasing out of high-energy-consuming and highly polluting enterprises, while high-tech and high-value-added enterprises progressively assume a dominant position, facilitating enhancements in carbon emission efficiency.

## Further analysis: The spatial spillover effects of IPDP

The empirical findings validate the significance of IPDP in improving carbon emission efficiency and elucidate the mechanisms through which IPDP impacts this efficiency. However, the potential spillover ramifications of the local implementation of IPDP on the carbon emission efficiency of adjacent regions have not been investigated. Hence, we propose developing a spatial econometric model to analyze these spatial spillover effects. By constructing spatial weight matrices, the model considers the spillover effects among cities in close geographical proximity and those with comparable levels of economic development, utilizing three distinct types of spatial weight matrices, as shown in the following.

5 $$W_{i,j}^{a} = \left\{ \begin{gathered} 0,i = j \hfill \\ \frac{1}{{d_{i,j}^{2} }},i \ne j \hfill \\ \end{gathered} \right.$$

where $W_{i,j}^{a}$ represents the geographic distance matrix, $d_{i,j}^{{}}$ indicates the geographic distance between city $i$ and city $j$.

6 $$W_{i,j}^{b} = \left\{ \begin{gathered} 0,i = j \hfill \\ \frac{1}{{\left| {GDP_{i} - GDP_{j} } \right|}},i \ne j \hfill \\ \end{gathered} \right.$$

where $W_{i,j}^{b}$ denotes the economic distance matrix, $GDP_{i}$ and $GDP_{j}$ represent the GDP of city $i$ and city $j$, respectively.

7 $$W_{i,j}^{c} = \left\{ \begin{gathered} 0,i = j \hfill \\ W^{\prime}{\text{*diag}}\left( {\frac{{\overline{GDP1} }}{{\overline{GDP} }},\frac{{\overline{GDP2} }}{{\overline{GDP} }}....\frac{{\overline{GDPi} }}{{\overline{GDP} }}} \right),i \ne j \hfill \\ \end{gathered} \right.$$

where $W_{i,j}^{c}$ represents the matrix that nests economic and geographic distances, $W^{\prime}$ is the geographic distance matrix, $\overline{GDP1}$ denotes the average annual GDP of city $i$, and $\overline{GDP}$ is the average GDP of all sample cities.

In addition, likelihood ratio (LR) tests were performed to determine the suitable spatial econometric model, incorporating geographical distance, economic distance, and a nested matrix of economic-geographical interactions, as depicted in Table 8. The analysis resulted in p-values of zero, indicating rejection of the null hypothesis. Consequently, adopting a Spatial Durbin Difference-In-Differences (SDM-DID) model was deemed appropriate. Following this, we constructed the SDM-DID model^50^.

8 $$CEE_{i,t} = \rho \sum W_{ij} CEE_{i,t} + \beta IPDP_{i,t} + \gamma \sum W_{ij} IPDP_{i,t} + \theta X_{i,t} { + }\varepsilon_{i,t}$$

where $\rho$ represents the spatial autoregressive coefficient, $\beta$, $\gamma$ and $\theta$ represent estimated coefficients within the statistical model.

**Table 8:** Suitability test for the spatial durbin model.

Type of test	Geographic distance matrix		Economic distance matrix		Economic-geographic distances	
	Statistical	*P* value	Statistical	*P* value	Statistical	*P* value
LR spatial lag	43.14	0.00	128.86	0.00	91.87	0.00
LR spatial error	22.75	0.00	144.23	0.00	104.79	0.00

Table 9 presents the regression outcomes from the SDM-DID model. The outcomes reveal that across three variations of spatial weight matrices, the spatial autoregressive coefficient $\rho$ demonstrates a statistically significant positive association, implying spatial autocorrelation within carbon emission efficiency. Additionally, in the indirect effects (utilized for assessing spatial spillover effects), the IPDP regression coefficients are significantly positive, indicating that the IPDP can generate spatial spillover effects on neighboring cities, consequently augmenting their carbon emission efficiency. More specifically, the intellectual property demonstration cities can play a model role within the region, generating knowledge spillover and diffusion effects, encouraging surrounding non-demonstration cities to learn from the demonstration cities, thereby enhancing their intellectual property protection frameworks, production techniques, and pollution control methods. Consequently, this process facilitates enhancing carbon emission efficiency in these cities.

**Table 9:** Results from the spatial durbin difference in difference model.

Variable	Geographic distance matrix			Economic distance matrix			Economic-geographic distances		
	Direct effect	Indirect effect	Total effect	Direct effect	Indirect effect	Total effect	Direct effect	Indirect effect	Total effect
IPDP	− 0.002 (0.033)	0.673*** (0.181)	0.671*** (0.196)	0.023 (0.032)	0.250*** (0.053)	0.273*** (0.071)	0.019 (0.033)	0.386*** (0.088)	0.405*** (0.097)
EDL	− 0.070*** (0.010)	− 0.126*** (0.023)	− 0.196*** (0.028)	− 0.092*** (0.010)	− 0.023*** (0.004)	− 0.114*** (0.012)	− 0.095*** (0.010)	− 0.040*** (0.007)	− 0.135*** (0.014)
EDL^2^	0.004*** (0.001)	0.007*** (0.001)	0.011*** (0.002)	0.005*** (0.001)	0.001*** (0.000)	0.006*** (0.001)	0.005*** (0.001)	0.002*** (0.000)	0.007*** (0.001)
ER	1.067 (2.905)	1.942 (5.470)	3.009 (8.337)	3.092 (2.955)	0.760 (0.747)	3.853 (3.690)	2.559 (2.890)	1.064 (1.241)	3.623 (4.117)
ES	− 0.169*** (0.044)	− 0.305*** (0.095)	− 0.473*** (0.132)	− 0.187*** (0.045)	− 0.047*** (0.013)	− 0.233*** (0.055)	− 0.187*** (0.045)	− 0.079*** (0.022)	− 0.266*** (0.064)
PSI	0.047 (0.073)	0.088 (0.139)	0.135 (0.211)	− 0.082 (0.077)	− 0.020 (0.019)	− 0.102 (0.095)	− 0.053 (0.074)	− 0.021 (0.032)	− 0.074 (0.106)
$\rho$	0.643*** (0.047)			0.207*** (0.028)			6.505*** (0.660)		
Observations	3640			3640			3640		

IPDP: Intellectual Property Demonstration Policies; EDL: Economic Development Level; EDL^2^: a quadratic term of economic development level; ER: Environmental Regulation; ES: Energy Structure; PSI: Proportion of Secondary Industry. The symbols *, **, and *** denote statistical significance at the 10%, 5%, and 1% levels, respectively, while the values in parentheses indicate standard errors.

7. Research conclusions and policy implications.

**Figure 5:**
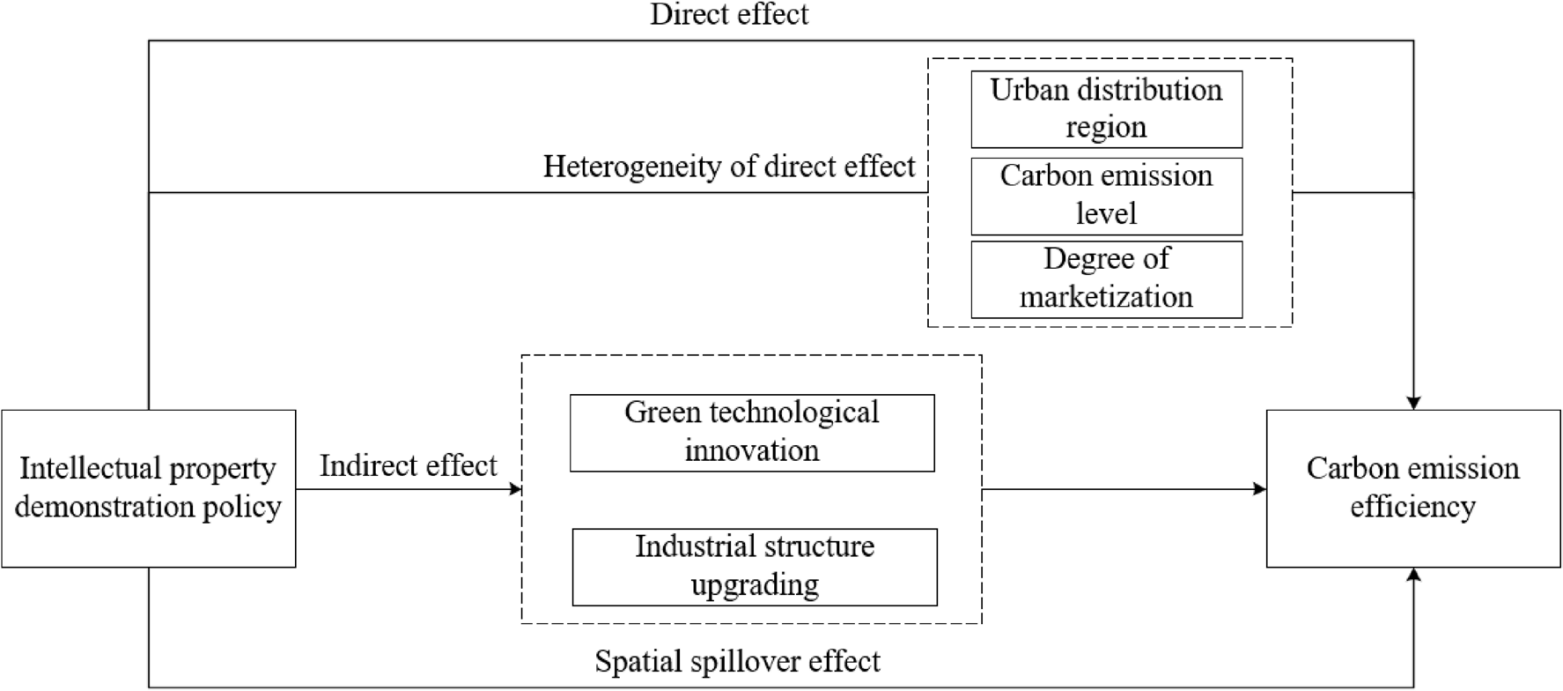
The Impact, Mechanism, and Spatial Spillover Effects of Intellectual Property Demonstration Policies on Carbon Emission Efficiency.

This study employs the IPDP as a quasi-natural experiment, utilizing annual data from 280 prefecture-level cities in China from 2007 to 2019. We constructed a multiple-time-point DID model, mediation effect model, and SDM-DID model to investigate the influence, mechanisms, and spatial spillover effects of IPDP on carbon emission efficiency, as depicted in Fig. 5. The findings reveal that: (1) Baseline regression analysis shows a significant enhancement of carbon emission efficiency due to IPDP implementation; (2) Heterogeneity tests demonstrate that the impact of the IPDP on carbon emission efficiency varies according to the urban distribution regions, carbon emission levels, and degree of marketization; (3) Mechanism tests suggest that the IPDP improves carbon emission efficiency through fostering green technological innovation and upgrading industrial structures; (4) Spatial spillover effect analysis confirms that the IPDP not only boosts local carbon emission efficiency but also promotes improvements in neighboring regions. These results underscore the efficacy of the IPDP in enhancing environmental outcomes through complex interplays of technological, structural, and regional dynamics.

The above research findings provide significant policy implications for accelerating the construction of China's intellectual property (IP) protection system to achieve green and low-carbon economic development. It recommends: (1) Further improvement of the IP system is necessary, continuing the selection and adjustment of IP demonstration cities in response to the “Intellectual Property Powerhouse Construction Outline (2021–2035)”. Specifically, it involves guiding market entities to capitalize on the synergistic effects of various types of intellectual property rights (e.g., patents, trademarks, copyrights), cultivating a group of globally competitive enterprises with robust IP capabilities, optimizing the IP management of national science and technology projects, and appropriately modifying IPDP to promote and endorse green and low-carbon patents. By encouraging the generation of green and low-carbon patents, cities can improve their overall carbon emission efficiency. Notably, it is imperative to consistently monitor the carbon emission efficiency of demonstration cities to promptly adjust the IP protection system, fully exploit the environmental benefits of IP demonstration policies, and significantly contribute to attaining carbon peak and carbon neutrality objectives. (2) Implement differentiated IP demonstration policies tailored to the unique characteristics and regions of cities, focusing on regional collaborative development. Accelerating the development of IP-intensive industries based on urban attributes and resource endowments is crucial. It involves fostering an innovation-driven economy supported by patents, a brand economy supported by trademarks, a characteristic economy supported by geographical indications, and a cultural tourism economy supported by copyrights. For instance, in the central and western regions, enhancing the geographical indication cultivation directory and exploring unique cultural resources like Yellow River culture, Silk Road culture, and ethnic culture can comprehensively enhance IP efforts, thereby aiding environmental sustainability. (3) Fully leverage the intermediary role of green technological innovation and industrial structure upgrading. Aligning IP protection with green innovation across different stages, implementing distinct policies and standards for the technology research and development and results transformation phases, and focusing on increasing the costs of infringing fundamental research outcomes are essential strategies. Optimizing the IPDP system by integrating green technological innovation in evaluating IP demonstration cities and reinforcing IP systems in ecological restoration and energy conservation sectors is crucial. These policies should moderately support emerging and knowledge-intensive industries, with local governments guiding diverse stakeholders to enhance support for these sectors, collaboratively developing new technologies, intensifying environmental penalties for high-energy-consumption and high-pollution industries, and expanding the share of technology-intensive industries in the industrial landscape. (4) Emphasize the spatial spillover effects of IPDP on neighboring areas to achieve coordinated improvements in carbon emission efficiency. The government should centrally coordinate efforts to promote intercity exchange and cooperation, establish networks for collaboration between IP demonstration cities and nearby cities, summarize the IP development experiences of demonstration cities, stimulate their spillover effects, and enhance their leading role in achieving development through demonstration.

Finally, this study has some limitations and several potential future directions. First, the measurement method for indicators may need to be sufficiently comprehensive. The study employs the Super-SBM model, which incorporates undesirable outputs to gauge this efficiency and encompasses input, desirable, and undesirable output factors. The undesirable output factor is represented by the total carbon emissions, calculated by summing the carbon emissions within each city's jurisdiction, the energy-related carbon emissions outside the jurisdiction, and emissions caused by internal activities outside the jurisdiction. However, this method may overlook some carbon emissions generated by urban activities. Therefore, in future research, we plan to manually collect more data on carbon emissions from internal and external urban activities to more comprehensively and accurately reflect the carbon emission efficiency indicators, thus making the research conclusions more reliable. Second, the heterogeneity tests need to be more comprehensive. While the research examines the heterogeneity of the impact of intellectual property demonstration policies on carbon emission efficiency in terms of regional, carbon emission, and marketization degree heterogeneity, additional grouping methods are needed to further investigate the diverse impacts of these policies on cities with varying characteristics. Third, the mechanisms covered need to be more comprehensive. Although the research primarily focuses on green technological innovation and industrial structure upgrading as the mechanisms through which intellectual property demonstration policies influence carbon emission efficiency, it acknowledges the complexity of these impact mechanisms. Future research intends to explore alternative perspectives to gain a more comprehensive understanding of how intellectual property demonstration policies influence carbon emission efficiency, thereby facilitating a more thorough investigation of the causal relationship between these policies and carbon emission efficiency.

## Data Availability

The authors will provide the raw data supporting the conclusions of this paper without reservation. Data could obtained directly from the corresponding author or the first author.
